# Glomangiomyoma of the neck in a child in Nepal: a rare case report and literature review

**DOI:** 10.1186/s12901-017-0041-0

**Published:** 2017-11-02

**Authors:** Bishow Tulachan, Buddha Nath Borgohain

**Affiliations:** Department of ENT - Head and Neck Studies, Universal College of Medical Sciences, Tribhuvan University Teaching Hospital, Bhairahawa, Nepal

**Keywords:** Glomangiomyoma, Glomus tumour, Angiography, Postoperative

## Abstract

**Background:**

Glomangiomyoma is a rare histological variant of glomus tumour. Clinically, it mimicks as a haemangioma and is challenging to diagnose. Its occurrence in the neck of a child has not been previously described.

**Case presentation:**

A 3 year old girl presented with the complaints of painless progressive neck swelling in the right side for one and half year. Sonography, computed tomography (CT), magnetic resonance imaging (MRI), CT neck angiography and fine needle aspiration cytology (FNAC) were suggestive of vacular malformation i.e. giant haemangioma or arteriovenous malformation. The mass was removed in toto under general anaesthesia without postoperative complications. The histopathology confirmed it to be glomangiomyoma with haemangiopericytoma like features.

**Conclusion:**

It’s an extremely rare variant of glomus tumour and may be the first report of a glomangiomyoma in the neck of a child. Despite a rare entity, it should be borne in mind during differential diagnosis.

## Background

Glomus tumour, an uncommon neoplasm, arises from the glomus bodies, cells having resemblance of the modified smooth muscle cells of the normal glomus body. Glomangiomyoma is a rare variant of it. Glomus bodies possess peculiar fibrous perivascular structures and regulate body temperature by functioning as arteriovenous shunts [1]. These are located in the reticular dermis throughout the body, especially in the sub ungual region, distal digits, and more acral portions of the body, but may occur wherever arteriovenous anastomoses are found [1–3]. However, these lesion are different from head and neck paragangliomas, which are also referred to as glomus tumours. Paragangliomas are tumours of the autonomic system arising from chromaffin cells of the parasympathetic paraganglia of the skull base and neck e.g. carotid body tumour [4].

In 1924, the first description about glomangiomyoma was given by Mason. It rarely occurs extradigitally particulary the neck region. And other extradigital sites where normal glomus bodies may be sparse or even absent, such as the patella, chest wall, bone, stomach, colon, nerve, eyelid, nose, mediastinum, small bowel, rectum, urinary tract, lung, cervix, vagina, oral cavity, mesentery, heart, lymph nodes, larynx, back and trachea have been reported [5–10]. Here, we report a case of glomangiomyoma of the neck in a 3 year old child. The histological and imaging findings are described.

## Case presentation

**Fig. 1:**
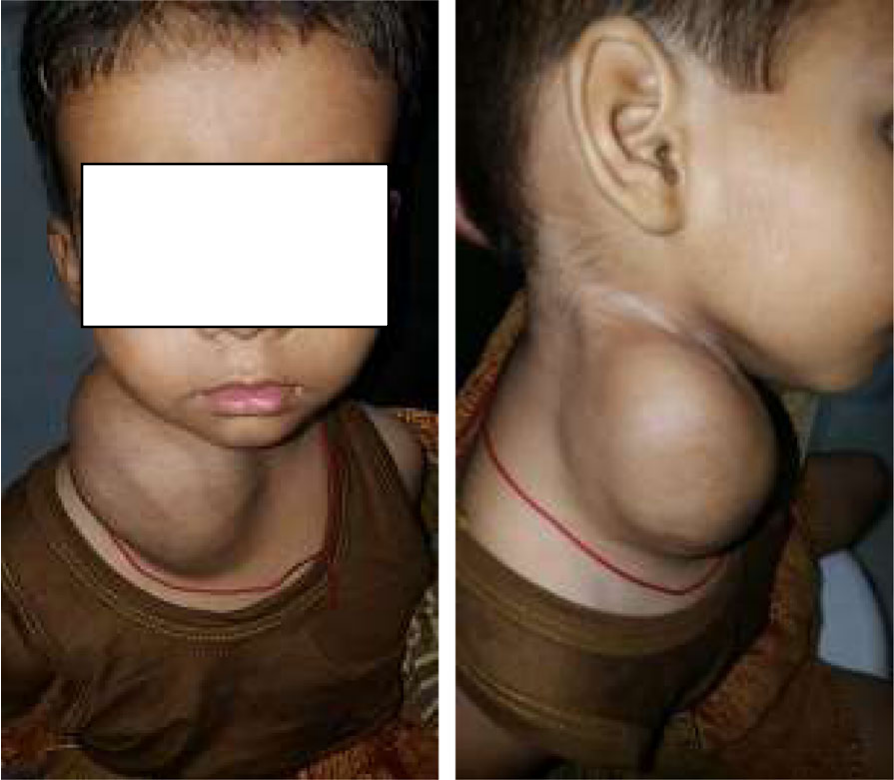
Anterior and lateral view of neck mass

This is a case report of a 3 year old female child presented with chief complaints of swelling in the right side of neck for around 1 ½ years in ENT OPD of Universal College of Medical Sciences, Bhairahawa, Lumbini zone. The onset was insidious and gradually progressive, painless and without aggravating and relieving factors. On examination, there was a 8 X 6 cm, ovoid, nontender, soft swelling, nonpulsatile, mobile in all directions, not fixed to overlying skin, smooth surface and prominent superficial neck veins and the transillumination test was positive. The swelling was extending superiorly at level of right angle of mandible, inferiorly 2 cm below the suprasternal notch, laterally 1 cm behind the posterior border of right sternocleidomastoid muscle and medially in the midline of neck (Fig. 1).

**Fig. 2:**
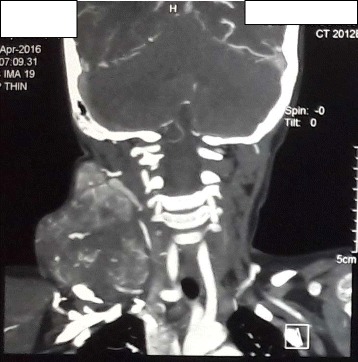
CT of neck (coronal view)

**Fig. 3:**
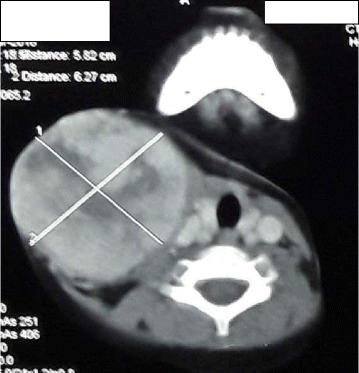
CT of neck (axial view)

**Fig. 4:**
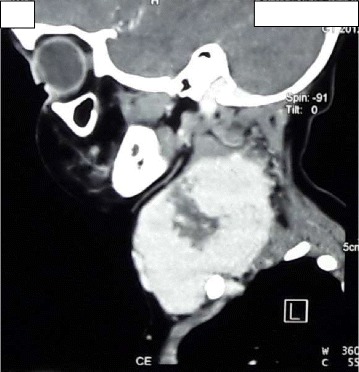
CT of neck (sagittal view)

**Fig. 5:**
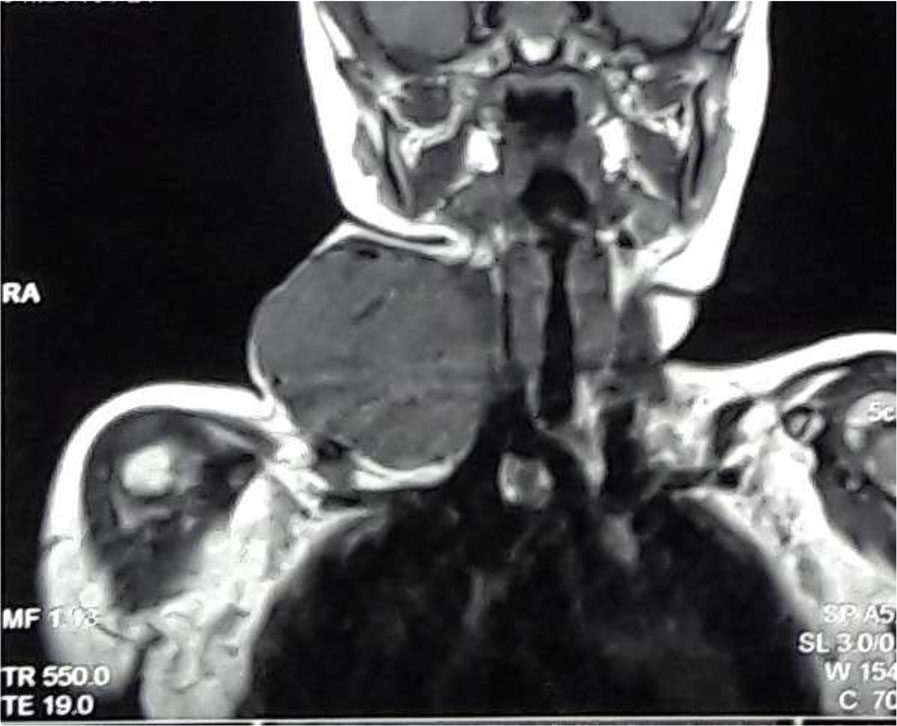
MRI of neck

The oral cavity, oropharynx and larynx examination were unremarkable. Hence, a provisional diagnosis of neck haemangioma/arteriovenous malformation was made. Ultrasonography with 9 MHz probe showed a large globular swelling nearly 10X8 cm filling right half of neck, multiple circumscribed primarily anechoic structures with appreciable fine internal echoes size varying from 0.87 to 3.67 cm, well marginated and the loculations are separated by thick intervening septa containing various sized vessels. Doppler couldn’t show flow in the swelling but the intervening tissue show larger blood vessels. Feeding vessel was not found and the whole large swelling was placed over the carotid sheath. FNAC showed only polymorphs, lymphocytes and macrophages in a background of RBCs suggestive of vascular lesion (Haemangioma). It was negative for malignancy. Plain and contrast enhanced CT was suggestive of high flow vascular malformation (Figs. 2, 3, 4). MRI was suggestive of high flow vascular malformation or neoplastic mass having high vascularity in it (Fig. 5).

**Fig. 6:**
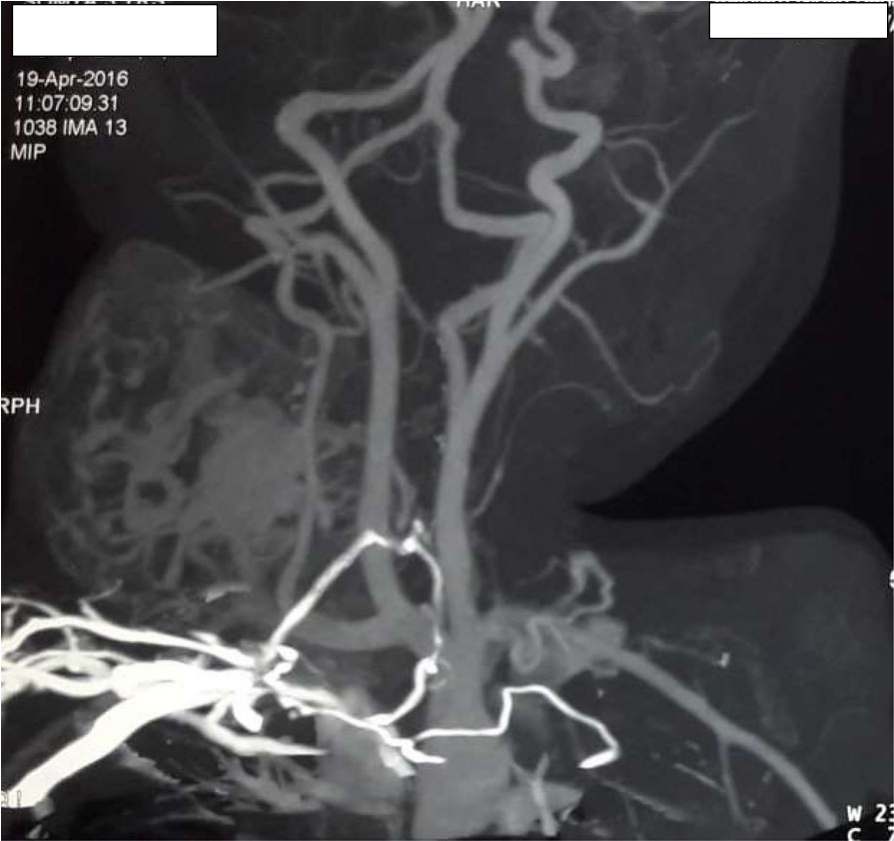
CT angiography of neck

**Fig. 7:**
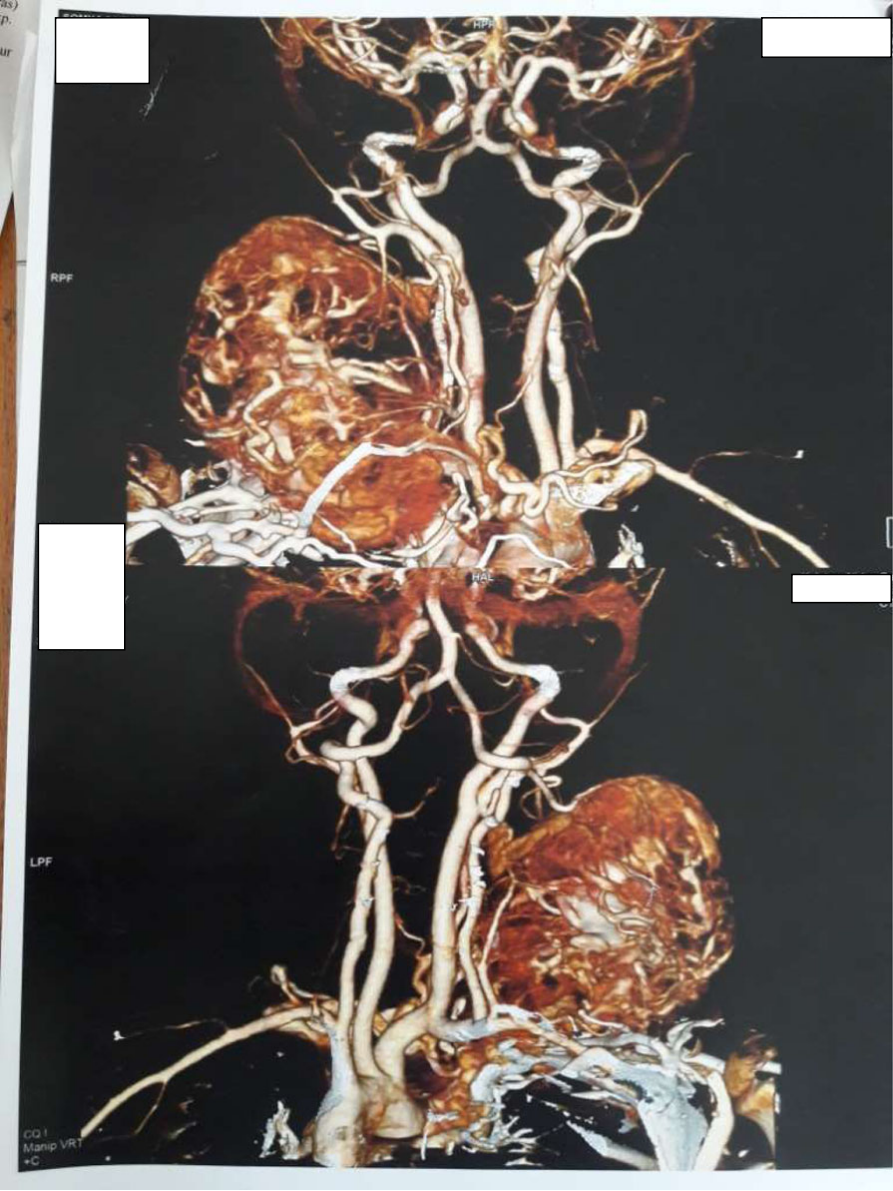
CT angiography (3D)

CT neck angiography was also suggestive of vascular malformation- giant haemangioma or arteriovenous malformation (AVM) (Figs. 6, 7).

**Fig. 8:**
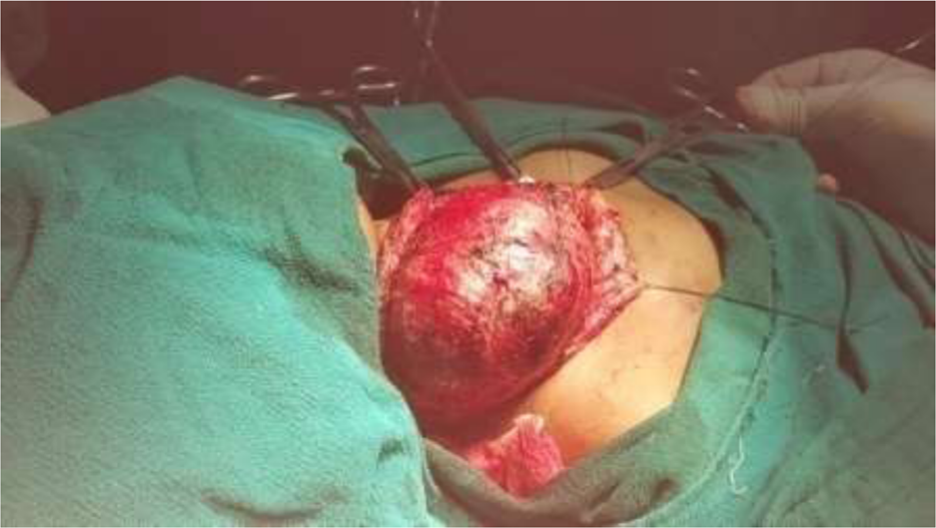
Exposure of mass with adherence to sternocleidomastoid and trapezius muscles

**Fig. 9:**
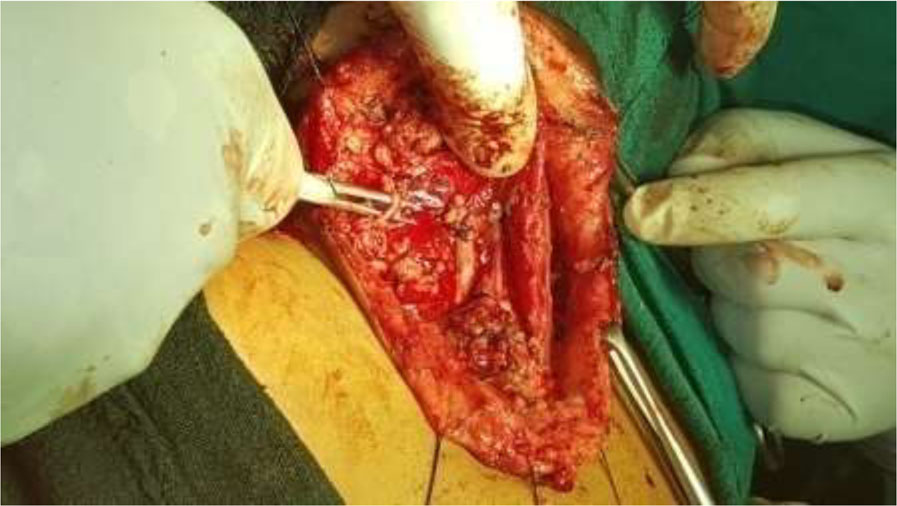
Accessory nerve

**Fig. 10:**
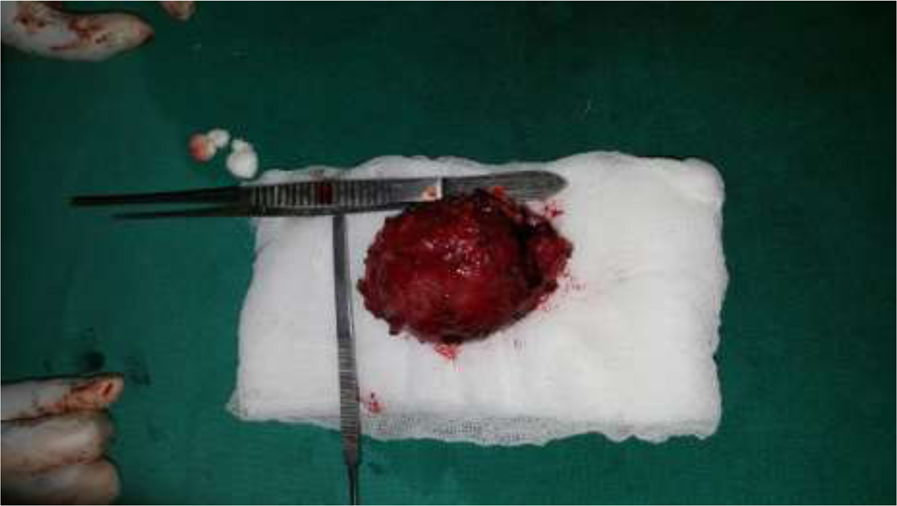
Excised mass 8 × 7 cm

**Fig. 11:**
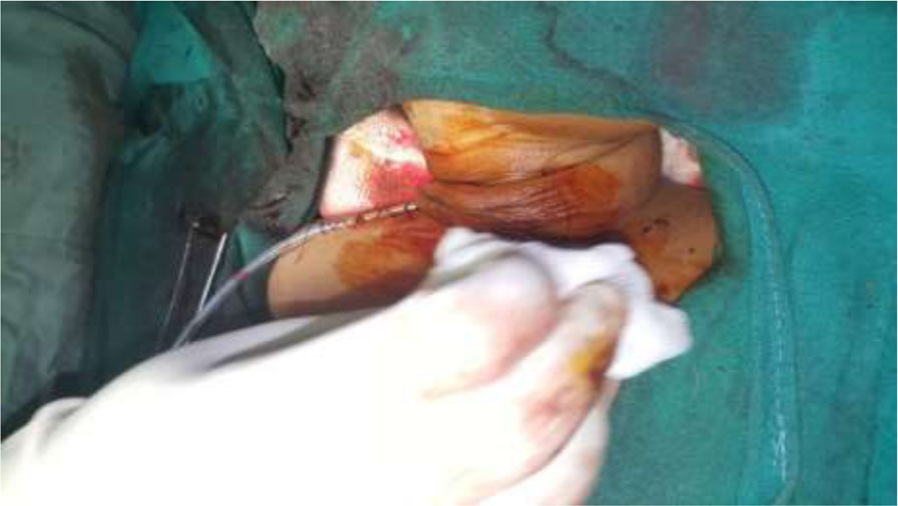
Skin closure with Prolene cutting body suture 4.0 and number 10 Romovac drain in situ

She underwent excision of the mass under general anaesthesia. Preoperative endovascular embolisation would have been better anticipating the blood loss during surgery but it’s not available locally. Hence, we solely depended upon the ligatures and electrocautery. Intraoperatively, the mass was adhered to sternocleidomastoid muscle extending upto hyoid bone level superiorly and extended inferiorly upto the supraclavicular fossa, feeding vessels from right subclavian, external carotid, common carotid artery, thyrocervical trunk were ligated and also the draining vessels towards internal jugular vein were also ligated and the entire mass was removed in toto (Figs. 8, 9, 10). Haemostasis was secured and closed with Romovac drain number 10 (Fig. 11).

**Fig. 12:**
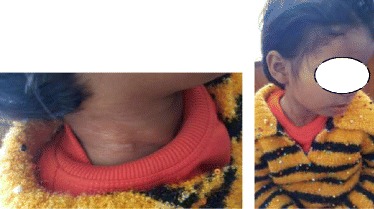
7 months follow up

**Fig. 13:**
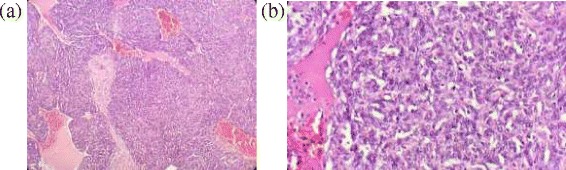
(**a**) low power view (×100) prominent thin walled blood vessels with proliferation of tumor cells around it along with variable proportion of smooth muscle and, (**b**) high power view (×400) individual tumor cells are small, uniform, round with central nucleus, discernible nucleoli and eosinophilic cytoplasm

Postoperatively she was free of complications and also at her subsequent follow ups (Fig. 12). Histopathology showed capsulated structure comprising of tumour cells arranged predominantly in solid sheets and nodules interrupted by variable sized vessels; many of which show staghorn like appearance. Few of the areas showed tumour cells arranged in nests and cords. Individual tumour cells revealed round to oval nuclei, bland dispersed nuclear chromatin, discernible nucleoli and moderate amount of cytoplasm. At the periphery of the sheets and nodules, many cells were spindled with elongated nuclei and bipolar eosinophilic cytoplasm resembling smooth muscle differentiation blended into tumour cells. Areas of necrosis were also evident. However, no overt atypia/atypical mitosis evident (Fig. 13). It was suggestive of perivascular tumour; glomangiomyoma with haemangiopericytoma like features.

## Discussion and conclusions

Glomus tumours are rare neoplasms, found typically in soft tissue of the extremities, notiably in the subungual region of the finger tip. However, extradigital identification have been done in different parts of the body [5, 6].Histologically, the tumour cells consists of varying proportions of glomus cells, vascular structures, and smooth muscle tissue. These are well-circumscribed lesions with tight convolutes of capillaries entangled by uniform glomus cells in a hyalinized or myxoid stromal background. Round and somewhat cohesive nature of the cells give them an epithelioid appearance. The histologic appearance of the tumors depends on the different factors like vascular cell–glomus cell ratio, their differentiation, and the amount and composition of the stroma. Solid glomus tumour (25%), glomangioma (60%) and glomangiomyoma are the recognized histological variants (15%) [7, 11]. Glomangiomyomas may have resemblance to that of an ordinary glomus tumour or a glomangioma. However, there’s a gradual trasition from glomus cells to elongated, mature smooth muscle cells. Immunohistochemically, glomus tumours show positive reactions for smooth muscle actin and CD34, and negative reactions for S-100 and cytokeratin [5]. Recently, glomuvenous malformations term was given to glomangiomas or glomangiomyomas. Glomuvenous malformations may either be acquired or congenital, and heterogenous germline mutations in the glomulin gene (GLMN) [12].

In our centre, it was diagnosed with the help of imaging like USG, contrast enhanced CT, CT angiography, MRI, FNAC and the histopathological evaluation.

The treatment of choice for glomus tumour is surgical excision. Several sclerosants like sodium tetradecyl sulphate, polidocanol and hypertonic saline has been reported to be effective. Ablative therapy with Argon and Carbon dioxide laser is of potential benefit for small, superficial lesions [12]. Here, we’ve described the clinical, radiological and the histopathological findings of a case of glomangiomyoma of the neck in a child. It can make the diagnosis difficult to other soft tissue tumours like haemangioma and av. malformations. However, it can be treated successfully with complete excision. To our knowledge, this may be the first report of a glomangiomyoma of the neck in a child.

## Abbreviations

AVM: Arteriovenous malformation
CT: Computed tomography
FNAC: Fine needle aspiration cytology
GLMN: Germline mutations in the glomulin gene
MRI: Magnetic resonance imaging
